# Novel Humanized Anti-HER3 Antibodies: Structural Characterization and Therapeutic Activity

**DOI:** 10.3390/antib14040084

**Published:** 2025-10-06

**Authors:** Alessia Muzi, Roberto Arriga, Giovanni Bulfaro, Francesca Fata, Antonella Costanzo, Valerio Chiarini, Manuela Cappelletti, Fabiana Fosca Ferrara, Federica Bucci, Linda Celeste Montemiglio, Carmelinda Savino, Emanuele Marra, Gennaro Ciliberto, Luigi Aurisicchio, Beatrice Vallone, Giuseppe Roscilli

**Affiliations:** 1 Takis s.r.l., 00128 Rome, Italy; arriga@takisbiotech.it (R.A.); chiarini@takisbiotech.it (V.C.); cappelletti@takisbiotech.it (M.C.); ferrara@takisbiotech.it (F.F.F.); bucci@takisbiotech.it (F.B.); marra@takisbiotech.it (E.M.); ciliberto@takisbiotech.it (G.C.); aurisicchio@takisbiotech.it (L.A.); 2 Department of Biochemical Sciences “Alessandro Rossi Fanelli”, Sapienza University of Rome, P. le Aldo Moro, 5, 00185 Rome, Italy; giovanni.bulfaro@uniroma1.it (G.B.); an.costanzo@uniroma1.it (A.C.); beatrice.vallone@uniroma1.it (B.V.); 3 Institute of Molecular Biology and Pathology, Department of Biochemical Sciences “Alessandro Rossi Fanelli”, Sapienza University of Rome, National Research Council, P. le Aldo Moro, 5, 00185 Rome, Italy; francescafata26@gmail.com (F.F.); lindaceleste.montemiglio@cnr.it (L.C.M.); carmelinda.savino@cnr.it (C.S.)

**Keywords:** humanized antibodies, tumor cell line, anti-tumor activity, HER3 receptor, X-ray crystallography

## Abstract

Background/Objectives: The ErbB protein family plays a critical role in the progression of various solid tumors, and HER3 has been implicated in resistance mechanisms to multiple cancer therapies due to its ability to form heterodimers with other ErbB receptors, thereby activating pathways that promote tumor growth and survival. This study aimed to generate and characterize humanized monoclonal antibodies against HER3 to inhibit its function and evaluate their potential as therapeutic agents. Methods: Murine monoclonal antibodies TK-A3 and TK-A4 were humanized and tested for binding to ErbB3 and competition with neuregulin-1β (NRG). Specificity was assessed by ELISA, and epitope identified by X-ray crystallography. Downstream signaling was analyzed by western blot for phosphorylated ErbB3, Akt, and MAPK. Antitumor activity was evaluated in vitro and in a pancreatic cancer xenograft model. A toxicology study was also conducted. Results: TK-hu A3 and TK-hu A4 bound specifically to ErbB3 without cross-reactivity to other ErbB receptors. The ErbB3-TK-hu A3 Fab structure revealed the binding epitope. Both antibodies competed with NRG, inhibiting ErbB3, Akt, and MAPK phosphorylation in a dose-dependent manner. They suppressed cancer cell survival in vitro, and TK-hu A3 significantly delayed tumor growth in vivo. The toxicology study indicated good tolerability. Conclusions: TK-hu A3 emerged as the lead candidate, showing specific HER3 targeting, strong pathway inhibition, and antitumor efficacy in vivo. Beyond standalone use, it could support novel strategies such as T-cell engagers, ADCs, CAR-T, and bispecific antibodies. These findings highlight TK-hu A3 as a promising therapy for HER3-positive, treatment-resistant cancers, meriting further development.

## 1. Introduction

The ErbB protein family, a subset of receptor tyrosine kinases (RTKs), includes four members: EGFR (ErbB1/HER1), HER2 (ErbB2), HER3 (ErbB3), and HER4 (ErbB4). In recent years, these receptors have been shown to play critical roles in the initiation and maintenance of several solid tumors. ErbB family members are frequently overexpressed in various cancers, including breast, colorectal, lung, head and neck, glioblastoma, and gastric cancers. Consequently, they are considered prime targets for approved small-molecule tyrosine kinase inhibitors (TKIs) and therapeutic monoclonal antibodies in clinical practice [1,2,3].

The activation of the ErbB family relies on the binding of extracellular ligands, which promote receptor dimerization and the activation of intracellular kinase domains. Unlike other family members, HER3 can only signal when it forms a heterodimer with other ErbB receptors due to its functionally inactive tyrosine kinase domain in the cytoplasmic portion [4]. While EGFR and ErbB2 are the main binding partners for HER3, it has also been shown to form heterodimers with the activated hepatocyte growth factor receptor (HGFR) [5] and fibroblast growth factor receptor 2 (FGFR2) [6]. Upon binding with one of its ligands, a neuregulin (NRG) such as NRG1 or NRG2, HER3 forms functional heterodimers, triggering autophosphorylation and enabling downstream signaling through pathways such as PI3K/AKT, MAPK, and JAK/STAT [7]. These pathways induce the expression of genes responsible for cancer cell proliferation, angiogenesis, migration, metastasis, and drug resistance [8,9].

Given this, it is reasonable to expect that the loss of HER3 activity may block cancer progression in systems driven by various RTKs. Studies have shown that small-interfering RNA (siRNA) knockdown of HER3 in ErbB2-amplified breast cancer cells can produce anti-proliferative effects similar to those achieved by ErbB2 siRNA knockdown, further underscoring the importance of HER3 and its interactions with other RTKs [10]. Additionally, somatic mutations of the HER3 receptor in several cancers, particularly in colon and gastric cancers, have been found to promote cellular transformation. However, these oncogenic mutations are dependent on ErbB2 activity, and their signaling can be blocked by targeted therapeutics [11].

Recent studies have highlighted the significance of HER3 in various cancer types. For instance, HER3 (ErbB3) mutations, while less frequent, play a critical role in the oncogenesis of certain tumors and are often associated with resistance to therapies targeting other ErbB family members [12]. Furthermore, NRG1 fusion-driven tumors have been shown to rely heavily on HER3 signaling, suggesting that targeting this pathway could be therapeutically beneficial [13,14].

Beyond directly promoting tumor growth, HER3 has also been implicated in conferring resistance to several targeted drugs, including EGFR inhibitors [15], ErbB2 inhibitors [16,17], and inhibitors of downstream kinases [18,19]. This dual role in driving both tumor growth and therapeutic resistance makes HER3 an attractive target for therapeutic intervention.

Recent advancements have renewed interest in HER3 as a therapeutic target, particularly due to its role in conferring resistance to existing cancer therapies. Chen et al. (2024) has delved into the molecular mechanisms by which HER3 contributes to cancer therapeutic resistance, highlighting the receptor’s ability to form a trimeric complex with ErbB2 and other receptors [20]. This trimeric complex plays a significant role in enhancing cancer cell survival and resistance to targeted therapies. The study further emphasizes the potential of novel therapeutic strategies aimed at disrupting this complex to overcome resistance and improve treatment outcomes [20]. The goal of this study is to generate a humanized IgG1 antibody against HER3 that can block cancer cell proliferation and tumor progression both in vitro and in vivo. In the present study, we examine the structural and functional characterization of the TK-hu A3 and TK-hu A4 variants derived from murine monoclonal antibodies. We report their binding to HER3 and demonstrate the competition with its ligand NRG. Binding specificity to other ErbB family members was evaluated using ELISA and epitope mapping. Moreover, we determined the crystallographic structure of HER3 in complex with TK-hu A3 Fab (PDB entry: 9I1Q) in order to reveal the interaction determinants and describe the epitope sequence in more detail.

## 2. Materials and Methods

### 2.1. Antibody Humanization

TK-A3 and TK-A4 mouse heavy and light chain sequences have been humanized following the germline grafting approach. For TK-A4, three variants of heavy and light chains were designed. For TK-A3, two variants were designed for the heavy chain and one for the light chain. In addition, following a minimalistic CDR grafting approach, one additional variant was designed for the heavy and light chains of murine TK-A3. The identified heavy and light variants were cloned into a mammalian expression vector in frame with the human IgG1 or kappa constant region present in the vector. All pairings between the designed variants of humanized heavy and light chains were then transfected in HEK293 cells.

### 2.2. Cell Culture

All cell lines were purchased from the American Type Culture Collection (ATCC, Manassas, VA, USA). The adenocarcinoma cell line MCF7, (code HTB-22) was maintained in Eagle’s Minimum Essential Medium (ThermoFisher Scientific, Waltham, MA, USA), supplemented with 0.01 mg/mL human recombinant insulin (ThermoFisher Scientific), and fetal bovine serum (FBS) to a final concentration of 10%. The adenocarcinoma cell line BxPC-3 (code CRL-1687) was maintained in RPMI-1640 medium (ThermoFisher Scientific) supplemented with 10% FBS. The squamous cell carcinoma FaDu, (code HTB-43), was maintained in Eagle’s Minimum Essential Medium (ThermoFisher Scientific), supplemented with 10% FBS. The adenocarcinoma cell line SK-BR-3 (code HTB30) was maintained in McCoy’s 5a Medium Modified (ThermoFisher Scientific), supplemented with 10% FBS. HEK293 cells (code CRL-1573) were maintained in DMEM (ThermoFisher Scientific) supplemented with 10% FBS. The Expi293-GnTI- cell line (code A39240, ThermoFisher Scientific) was maintained in Expi293 Expression Medium (ThermoFisher Scientific).

### 2.3. Transient Transfection and Production of Recombinant Antibodies in HEK293 Cells

Briefly, HEK293 cells were seeded into 10 cm plates until they reached 70–80% confluence. For each transfection sample, a total of 24 µg of DNA and 60 µL Lipofectamine 2000 (ThermoFisher Scientific, Waltham, MA, USA) were separately diluted in 1.5 mL of Opti-MEM™ Reduced Serum Medium (ThermoFisher Scientific). DNA and Lipofectamine solution were mixed and incubated for 15–30 min at room temperature (RT). DNA/Lipofectamine complexes were distributed over the cells and incubated for 24 h. Next day, the medium was completely replaced by DMEM supplemented with 10% (*v/v*) fetal bovine serum (FBS) (ThermoFisher Scientific) and penicillin/streptomycin (ThermoFisher Scientific). The medium was collected after 72 h and tested for binding to human HER3 by ELISA assay. The heavy and light chain combinations, which gave a higher signal in ELISA, were then produced in larger quantities and purified by Protein A affinity chromatography. Before purification, supernatant samples (25 µL each) were mixed with Loading buffer containing 1 mM dithiothreitol (DTT) and heated at 95 °C for 5 min to ensure reduction of disulfide bonds. Samples were then separated on 10% SDS-PAGE gels under reducing conditions. Gels were stained with Coomassie Brilliant Blue to assess antibody expression and integrity.

### 2.4. ELISA Analysis

ELISA assays were performed using plates coated with 1 µg/mL of the following proteins: human HER3-Fc chimeric protein (R&D Systems, Minneapolis, MN, USA); human HER3/HER3 (HisTag) (Sinobiological, Wayne, PA, USA); Rhesus HER3/HER3 (HisTag) (Sinobiological, Wayne, PA, USA); Rat HER3/HER3 (HisTag) (Sinobiological); Mouse HER3/HER3 (HisTag) (Sinobiological); Recombinant human HER3/HER3-Fc chimeric protein (R&D Systems, Minneapolis, MN, USA); Recombinant human EGFR-Fc chimeric protein (R&D Systems); Recombinant human ErbB2 chimeric protein (R&D Systems); Recombinant human ErbB4 chimeric protein (R&D Systems). The plates were then blocked with BSA3% in PBST (Phosphate-Buffered Saline + 0.05% Tween 20), incubated with supernatants from transfected cells, and detected with alkaline phosphatase (AP) conjugated anti-human IgG (Merck KGaA, Darmstadt, Germany) or anti-murine IgG (Merck KGaA). Alkaline phosphatase yellow (pNPP) liquid substrate (Merck KGaA) was added before the plates were analyzed on a VICTOR plate reader (PerkinElmer, Waltham, MA, USA).

### 2.5. Epitope Mapping

NuncMaxiSorp plates (ThermoFisher Scientific, Waltham, MA, USA) were coated with 1 µg/mL of 15 amino acid peptides with 11 amino acid overlaps (JPT Peptide Technologies GmbH, Berlin, Germany) and covering the entire HER3 protein in 50 mM carbonate buffer (pH 9.6), 100 µL per well, and incubated overnight at 4 °C. Plates were washed 3 times with PBST and then blocked in BSA 3%, 200 µL per well, 1 h at RT. TK-hu A3 and TK-murine A3 antibodies at a concentration of 1 µg/mL in BSA1%-PBST were incubated overnight at 4 °C. After 3 washes in PBST, the plates were incubated with the secondary anti-human IgG antibody (Fab specific)-peroxidase conjugated (Merck KGaA, Darmstadt, Germany) produced in rabbit and the anti-mouse IgG antibody (whole molecule)-alkaline phosphatase conjugated (Merck KGaA), both diluted 1:2000 in BSA1%-PBST for 1 h at 37 °C. After a further 3 washes in PBST, the plates were developed with alkaline phosphatase yellow substrate (pNPP) (Merck KGaA) and the absorbance was measured by a VICTOR plate reader (PerkinElmer, Waltham, MA, USA).

### 2.6. HER3-ECD Deglycosylation

The extracellular domain of human HER3 protein tagged with 6xHis tag, expressed in Expi293-GnTI- cells, was fully deglycosylated using the EndoH_f_ enzyme (New England Biolabs, Ipswich, MA, USA). The protein was first dialyzed in a buffer containing 20 mM Tris pH 8.0 and 150 mM NaCl. It was then incubated overnight with the EndoH_f_ enzyme, adding GlycoBuffer (0.5 M Sodium Acetate pH 6.0) to a final dilution of 1:10. The following day, the sample was purified from the EndoH_f_ enzyme during an overnight incubation at 4 °C using pre-equilibrated PureCube 100 Ni-NTA agarose resin (Cube Biotech, Monheim am Rhein, Germany). The equilibration was performed using a buffer containing 50 mM NaH_2_PO_4_, 300 mM NaCl, and 5 mM Imidazole at pH 8.0. Finally, the sample was centrifuged at 1300 rpm, and the supernatant containing the EndoH_f_ enzyme was discarded. The resin was washed with a buffer at pH 8.0 containing 50 mM NaH_2_PO_4_ and 300 mM NaCl, and the fully deglycosylated protein was eluted using a buffer containing 50 mM NaH_2_PO_4_, 300 mM NaCl, and 300 mM Imidazole pH 8.0. The eluted fractions were evaluated by SDS-PAGE and stained for glycosylation using the Pierce™ Glycoprotein Staining Kit (ThermoFisher Scientific, Waltham, MA, USA), and subsequently pooled.

### 2.7. TK-hu A3 Fab Fragments Generation and HER3::TK-hu A3 (Fab) Complex Crystallization

The monoclonal antibody TK-hu A3-H1L1 was digested using papain-conjugated resin (ThermoFisher Scientific, Waltham, MA, USA). Prior to digestion, the antibody was dialyzed against 20 mM NaH_2_PO_4_/Na_2_HPO_4_ containing 5 mM EDTA (pH 7.0). The dialyzed sample was then incubated overnight at 37 °C with the papain-conjugated resin. The following day, the mixture of Fc and Fab fragments, along with any undigested antibody, was incubated overnight with ToyoScreen AF-rProtein A HC-650F resin (Tosoh Bioscience, Tokyo, Japan), pre-equilibrated in 20 mM NaH_2_PO_4_/Na_2_HPO_4_ (pH 7.0). After incubation, the sample was centrifuged, and the supernatant, containing the Fab fragments, was collected. Undigested antibody and Fc fragments were subsequently eluted with 100 mM citrate buffer (pH 3.0).

For crystallization trials, deglycosylated HER3 was mixed with TK-hu A3-H1L1 Fab fragments at a 1:2 stoichiometric ratio (to ensure Fab excess) and incubated for 1 h at 4 °C. The mixture was then loaded onto a Superdex 200 Increase 10/300 GL size-exclusion chromatography column pre-equilibrated with 150 mM NaCl and 10 mM Tris-HCl (pH 8.0). The column was operated at a flow rate of 0.5 mL/min, and fractions corresponding to the HER3::TK-hu A3 (Fab) complex were pooled and concentrated to 10 mg/mL. Protein concentrations (HER3 ECD, Fab and SEC-purified complex) were determined by UV absorbance at 280 nm using sequence-derived extinction coefficients.

Crystallization was performed by sitting-drop vapor diffusion at 20 °C with 1:1 protein:reservoir drops (0.4 µL total) equilibrated against a 35 µL reservoir. Initial sparse-matrix screening was set up on an Oryx4 robot (Douglas Instruments, Hungerford, UK) in 96-well sitting-drop plates and identified the following successful condition: 0.2 M KSCN, 0.1 M BIS-TRIS propane (pH 8.5), and 20% (*w/v*) PEG 3350. Optimization followed a serial microseeding workflow: (i) seed stock S1 was prepared from the initial robot-generated sitting-drop crystals; using hand-made hanging drops in 24-well plates (total drop volume 2 µL equilibrated against a 500 µL reservoir; typical protein:reservoir:seed = 1:0.5:0.5) with S1 yielded improved crystals; (ii) a second seed stock S2, prepared from those improved crystals, was then used in hand-made 24-well hanging drops to obtain the final diffracting crystals used for data collection.

Across seeding rounds, the PEG 3350 concentration was varied around the initial hit to tune nucleation versus growth. Seed stocks were obtained by collecting crystals from the drops, suspending them in mother liquor, crushing, storing at −80 °C, and diluting 1:10, 1:100, or 1:1000 immediately before set up.

### 2.8. Data Collection

Data collection was performed on crystals obtained from the second phase of microseeding in the condition of 0.2 M KSCN, 0.1 M BIS-TRIS propane pH 8.5, and 14% PEG 3350 with seeds diluted 1:100. The crystals grew within 24 h. X-ray diffraction data for the HER3::TK-hu A3 Fab complex were collected at the ELETTRA synchrotron (Trieste, Italy). The dataset was collected at 100 K using a PILATUS detector and processed with the STARANISO software [21]. The data collection statistics are summarized in Table 1.

The crystal structure was solved using the molecular replacement method with PhaserMR [22], implemented in the CCP4 suite [23]. The chain C of the 4LEO structure [24], which corresponds to the inactive state of HER3, and the TK-hu A3 Fab model predicted using the AlphaFold2 software [25] were used as the search model. Iterative automated structure refinement and manual model building were carried out using the Phenix suite [26] and COOT [27]. For copy-to-copy comparison, models were least-squares superposed on HER3 Cα atoms over residues 192–219 in UCSF ChimeraX v1.9 ([28] in supplementary), and pairwise Cα RMSDs relative to chain B (1.03/1.63/1.55 Å for B–A/B–C/B–D) were reported (Supplementary Figure S2). Interface residues were defined with PDBePISA and curated using a ~4.0 Å heavy-atom baseline with chemical-plausibility filtering (see Results); B-factor maps (blue–white–red, 9–262 Å^2^; white = 85 Å^2^) were used to compare interface disorder across ASU copies (Supplementary Figure S3). Coordinates and structure factors have been deposited in the PDB under accession 9I1Q.

### 2.9. Flow Cytometry-Based Binding Assay

2 × 10^5^ cells/tube of MCF7 or SK-BR-3 were incubated on ice for 1 h with different concentrations of TK-murine A3, TK-hu A3 and TK-hu A4 antibodies with or without NRG at 100 ng/mL in 100 mL of Facs Buffer (PBS1X + 0.5 mM EDTA + 0.1%FBS). Cells were washed, and antibody binding was detected with anti-mouse or anti-human IgG antibody-Alexa Fluor 488 (ThermoFisher Scientific, Waltham, MA, USA). The Allophycocyanin (APC) conjugated anti-human HER3/HER-3 Antibody (BioLegend, San Diego, CA, USA) was used as positive control. Cells were then analyzed by CytoFLEX flow cytometer platform (Beckman Coulter, Brea, CA, USA).

### 2.10. Antibody Binding Affinity

The interactions of HER3 Fc chimera protein (R&D Systems, Minneapolis, MN, USA) with TK-murine A3, TK-murine A4, TK-hu A3, and TK-hu A4 antibodies were studied by surface plasmon resonance detection using a Biacore X100 instrument (Cytiva, Marlborough, MA, US). HER3 Fc chimera protein was coupled to a research-grade carboxymethylated dextran sensor chip (CM5, Biacore, Uppsala, Sweden). Kinetic analysis was performed employing the single-cycle kinetics assay, in order to avoid the extensive use of the regeneration procedure that is detrimental to the ligand or the multiple kinetics assay. The analytes were injected from low to high concentration with 180 s contact time and 600 s dissociation times in between. Injections were performed at 25 °C with a flow rate of 30 mL/min. The sensor chip surface was regenerated with 10 mM glycine/HCl, pH 2.0, for 30 s, unless otherwise specified. All the experiments were performed in duplicate, and the reported *k*_on_ and *k*_off_ values are the averages of values arising from the two experiments, while *K*_D_ = *k_off_/k*_on_.

### 2.11. Antibody Treatments and Immunoblot Analysis

MCF7, FaDu, or BxPC-3 cells were seeded at 0.3 × 10^6^ per well. The following day, the medium was removed, and cells were treated or not with antibodies at the indicated concentrations for 6 h. 15 min before the end of treatment, Neuregulin-1beta (R&D Systems) was added at a concentration of 100 ng/mL to activate the HER3 signal pathway. Thereafter, the cells were washed once with ice-cold PBS and then lysed by adding RIPA buffer (Merck KGaA, Darmstadt, Germany). After a brief incubation, cell lysates were collected and equal amounts were loaded onto NuPAGE Novex Bis-Tris gels (ThermoFisher Scientific, Waltham, MA, USA), and proteins were transferred onto polyvinylidene fluoride membranes (ThermoFisher Scientific). Membranes were blocked with 5% nonfat dry milk and 0.1% Tween 20 (Merck KGaA) in Tris-buffered saline (TBS), pH 7.4, and incubated overnight at 4 °C with antibodies against phospho HER3-Tyr1289 (code #2842, Cell Signaling Technology, Danvers, MA, USA), phospo AKT-Ser473 (code #4060, Cell Signaling Technology), Phospho-p44/42 MAPK (Erk1/2) (Thr202/Tyr204) (code #4377, Cell Signaling Technology), HER3 (code #4754, Cell Signaling Technology, ), AKT (code #4691, Cell Signaling Technology), p44/42 MAPK (Erk1/2) (code #4695, Cell Signaling Technology). An antibody against β-tubulin (code sc-5274, Santa Cruz Biotechnology) was used to ensure loading of equal amounts of protein across all wells. Membranes were washed in TBS containing 0.1% Tween 20 and then incubated for 1 h with the anti-rabbit IgG horseradish peroxidase-conjugated streptavidin secondary antibodies (code #7074, Cell Signaling Technology) for all Cell Signaling antibodies and with anti-mouse IgG (whole molecule)-Peroxidase conjugated antibody (Merck KGaA, Darmstadt, Germany) for β-tubulin. After washing, protein bands were detected using SuperSignal West Femto Chemiluminescent substrate or SuperSignal West Pico Chemiluminescent substrate (ThermoFisher Scientific, Waltham, MA, USA). Images were acquired using the Chemidoc system (Bio-Rad, Hercules, CA, USA).

### 2.12. Colony-Formation Assay

BxPC-3 cells were seeded in triplicate into a 6-well plate (5 × 10^2^/well), and after overnight incubation, treated with three different antibodies (TK-murine A3, TK-hu A3-H1L1 and TK-hu A4-H3L1) at the scalar doses of 100 µg/mL, 50 µg/mL, 25 µg/mL, 10 µg/mL, and 1 µg/mL. Cells were cultured for colony-formation. After 10–14 days, colonies were fixed, stained with 2% methylene blue in 95% ethanol, and counted. Only colonies comprising >50 cells were scored as survival colonies.

### 2.13. Proliferation Assay

Proliferation assay was performed using CellTiter-Glo Luminescent Cell Viability Assay (Promega, Madison, WI, USA). Briefly, 7000 cells/well were seeded in triplicate and treated with different concentrations of TK-hu A3-H1L1 and TK-hu A4-H3L1 (100 µg/mL, 50 µg/mL, 25 µg/mL, 10 µg/mL, and 1 µg/mL). After 72 h, the number of metabolically active cells was determined based on levels of the ATP present, according to the following reaction: mono-oxygenation of luciferin is catalyzed by luciferase in the presence of Mg^2+^, ATP and molecular oxygen. Luminescence was recorded using a VICTOR plate reader (PerkinElmer, Waltham, MA, USA).

### 2.14. In Vivo Study

All studies have been performed in accordance with the “Directive 2010/63/EU on the protection of Animals used for scientific purposes” that was made effective in Italy by the Legislative Decree DLGS 26/2014. We used 12-week-old male nude mice (Charles River Laboratories, Lecco, Italy). Mice were maintained in laminar flow cages with sterile food and water, ad libitum. 1 × 10^6^ BxPC-3 cells were inoculated subcutaneously into the right flank of mice and treatment started when tumors reached an average size of 100 mm^3^. The mice were intraperitoneally (i.p.) injected with 20 mg/kg of TK-hu A3-H1L1 and TK-hA4-H3L1, twice a week, till control tumors reached the average size of 1500 mm^3^. The tumor size was measured with a linear caliper twice a week up to 4 weeks, and the volume was estimated using the equation V = (a × b^2^)/2, where a is the length of the major axis and b is the length of the minor axis.

### 2.15. Toxicity Study of TK-hu A3-H1L1 in Rats

Six males and six females were treated with a single dose of TK-hu A3-H1L1, either at 20, 40, or 80 mg/kg dose levels, i.p. The administration volume was 20 mL/kg. A control group of 3 animals/gender was also set up that received a single i.p. injection of 20 mL/kg of the vehicle (sterile PBS, pH 7.4). The antibody was diluted in sterile PBS immediately prior to the dose, to obtain the required concentrations. Formulated TK-hu A3-H1L1 and sterile PBS were administered to each animal via single-use sterile syringes. Clinical observations, mortality, and body weight were recorded during the study. At the end of the experiment (day 5), all the animals were bled in fasting condition, under isofluorane anesthesia, for the hematochemical analysis, sacrificed by CO_2_ asphyxia, and finally submitted for pathological examinations.

### 2.16. Statistical Analysis

Data are expressed as mean ± SD. Results were analyzed by paired t-test and *p*-value of less than 0.05 were considered statistically significant. Interventionary studies involving animals or humans, and other studies that require ethical approval must list the authority that provided approval and the corresponding ethical approval code. In this section, where applicable, authors are required to disclose details of how generative artificial intelligence (GenAI) has been used in this paper (e.g., to generate text, data, or graphics, or to assist in study design, data collection, analysis, or interpretation). The use of GenAI for superficial text editing (e.g., grammar, spelling, punctuation, and formatting) does not need to be declared.

## 3. Results

### 3.1. Antibody Humanization

**Figure 1:**
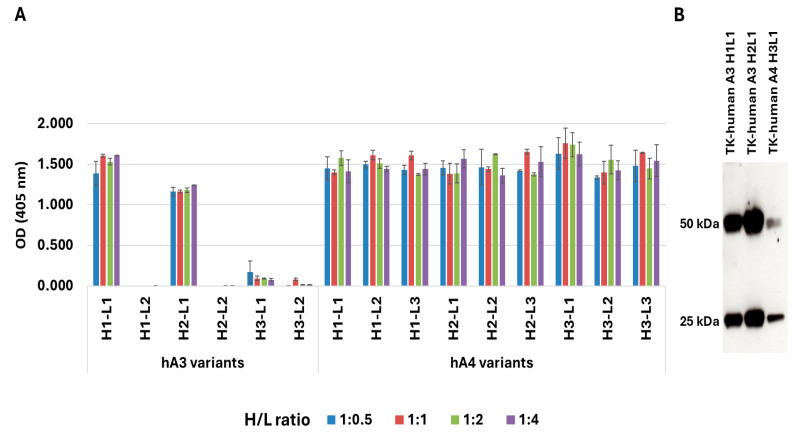
Identification of variants binding HER3. (**A**) Binding activity of TK-hu A3 and TK-hu A4 antibody variants to human HER3, as measured by ELISA. Six combinations of TK-hu A3 heavy (H) and light (L) chains and nine combinations of TK-hu A4 variants were transiently expressed in HEK293 cells. Supernatants were harvested 72 h post-transfection and assessed for HER3 binding. Bars represent mean absorbance ± SD of triplicate measurements. H/L ratio: DNA heavy chain to DNA light chain ratio. (**B**) Expression and integrity of recombinant mAbs H1L1 (lane 1), H2L1 (lane 2) from TK-hu A3, and H3L1 (lane 3) from TK-hu A4 were analyzed by SDS-PAGE under reducing conditions (1 mM DTT) followed by Coomassie blue staining. Molecular weight markers corresponding to the heavy chain (~50 kDa) and light chain (~25 kDa) are indicated.

The murine monoclonal antibodies TK-A3 and TK-A4 were humanized using a germline CDR grafting approach, as detailed in the methods. For TK-A4, three variants of heavy and light chains were designed, while TK-A3 had two variants for the heavy chain and one for the light chain, with an additional variant designed following a minimalistic CDR grafting approach. These variants were cloned into mammalian expression vectors and transfected into HEK293 cells to identify the heavy and light chain pairs that were correctly folded and secreted. The supernatants from transfected cells were analyzed by ELISA for their ability to bind the HER3 antigen (Figure 1A). For TK-hu A3, only two out of six combinations, specifically H1L1 and H2L1, successfully bound the antigen. For TK-hu A4, all variants detected the antigen, with H3L1 showing the highest signal, as indicated by the ELISA readings. These three variants (H1L1 and H2L1 from TK-hu A3, and H3L1 from TK-hu A4) were produced in larger quantities and purified by Protein A affinity chromatography. Coomassie blue staining (Figure 1B) confirmed that all three combinations correctly expressed and secreted both heavy and light chains, although TK-hu A4 exhibited lower expression levels compared to TK-hu A3.

### 3.2. Species Cross-Reactivity

**Figure 2:**
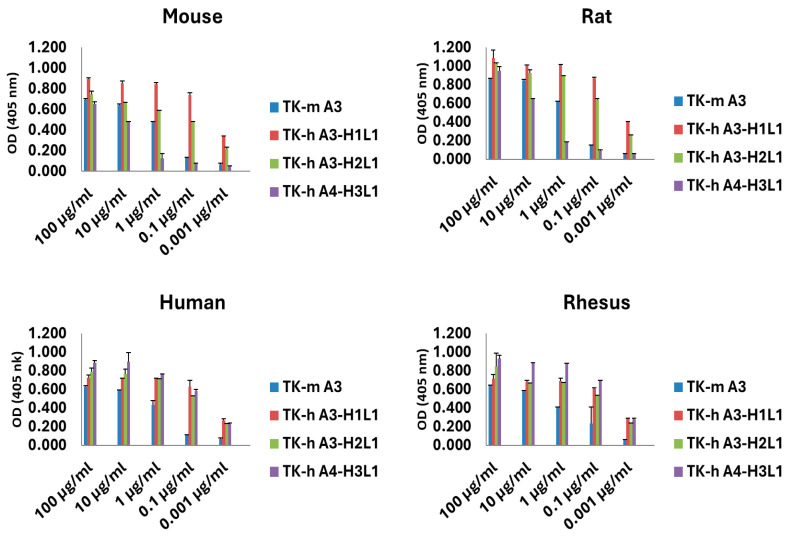
Antibodies species cross-reactivity. Binding of TK-murine A3, TK-hu A3, and TK-hu A4 purified antibody variants to recombinant mouse, rat, human, and rhesus HER3 was determined in a dose–response experiment by ELISA. Data represent mean absorbance ± SD from triplicate wells.

The species cross-reactivity of our antibodies was assessed in a dose–response experiment by ELISA, utilizing recombinant proteins from human, mouse, rat, and rhesus HER3. The results demonstrated comparable binding of the antibodies to HER3 from all species tested (Figure 2), indicating that these antibodies are suitable for use in preclinical animal models. This cross-reactivity supports the use of these antibodies in pharmacokinetics, toxicity, and safety studies across various species, including rodents and non-human primates.

### 3.3. ErbB Family Receptor Specificity for TK-hu A3 and TK-hu A4

To determine whether humanization affects ligand specificity, we analyzed the binding activity of the purified humanized monoclonal antibodies (mAbs) against recombinant proteins from other receptors of the ErbB family. Among the EGFR family receptors, HER2, HER3, and HER4 are closely related and share relatively high sequence and structure similarity [29]. We then assessed whether humanized A3 and A4 could cross-react with other EGFR family members.

**Figure 3:**
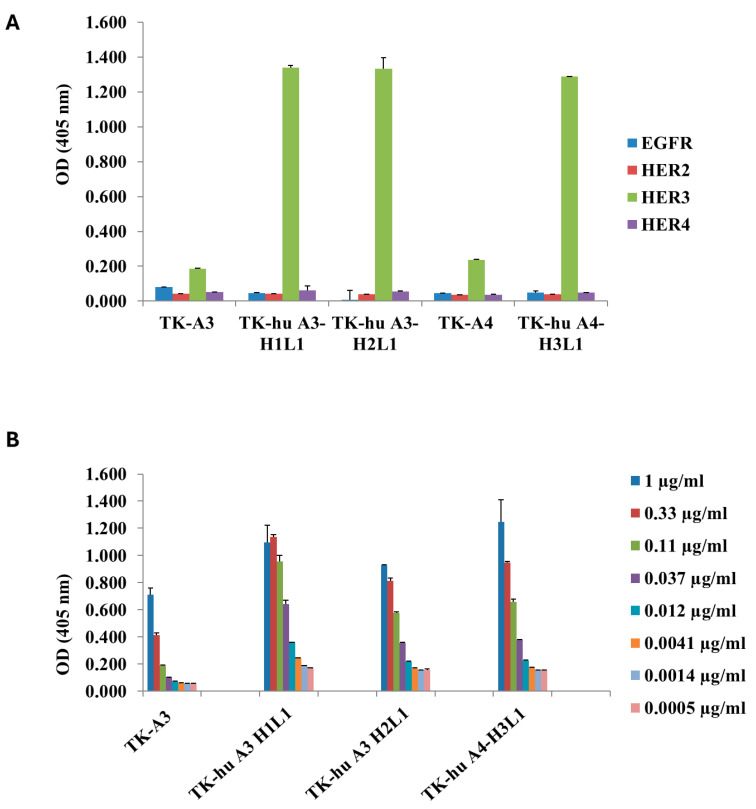
Binding activity of TK-hu A3 and TK-hu A4 antibody variants. (**A**) TK-murine A3 and TK-murine A4 antibodies, as well as TK-hu A3 and TK-hu A4 purified antibody variants, were analyzed for their binding to members of the ErbB tyrosine kinase receptor family: EGFR, ErbB2, HER3, and ErbB4. Antibodies were used at a concentration of 10 µg/mL, and binding was analyzed by ELISA. Absorbance values are plotted on the y-axis with respect to the antibody tested on the different recombinant proteins. (**B**) Serial dilutions of TK-murine A3, TK-hu A3, and TK-hu A4 purified antibody variants were analyzed by ELISA to assess their binding to human HER3 in a dose-dependent manner. Absorbance values are plotted on the y-axis with respect to the antibody tested at different dilutions.

The results demonstrated that antibody humanization did not interfere with the specificity of the selected antibodies, as the humanized variants did not recognize EGFR, ErbB2, or ErbB4, as determined by ELISA (Figure 3A). The purified antibodies were also tested in a dilution series to assess their binding affinity to HER3, demonstrating that all three antibodies (H1L1 and H2L1 from TK-hu A3, and H3L1 from TK-hu A4) retained a binding capacity similar to the parental murine antibodies, maintaining the same apparent affinity (Figure 3B).

### 3.4. Epitope Mapping

**Figure 4:**
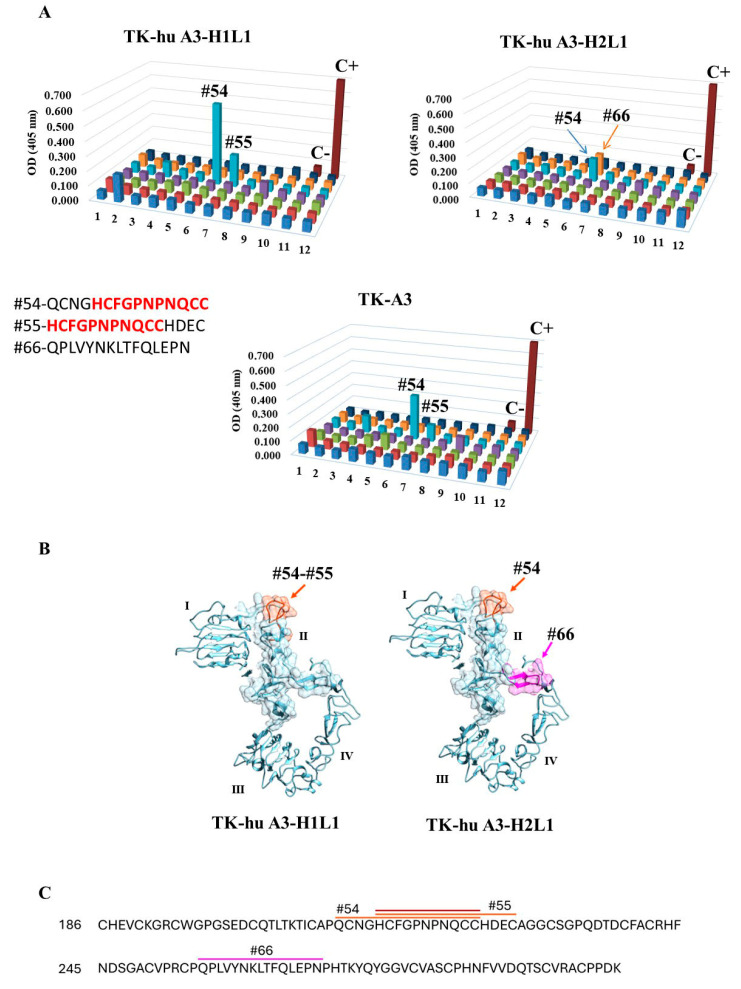
Epitope mapping of murine and humanized anti-HER3 antibodies. (**A**) Peptide array binding assays for TK-hu A3-H1L1, TK-hu A3-H2L1, and the murine antibody TK-A3. Reactivity was measured by ELISA using overlapping 15-mer peptides covering the HER3 extracellular domain (ECD). Each colored bar represents the optical density (OD) at 405 nm for an individual peptide. Strong binding was observed for peptides #54 and #55 in TK-hu A3-H1L1 and TK-A3, indicating conserved epitope recognition. In contrast, TK-hu A3-H2L1 showed loss of binding to these peptides and gained reactivity for peptide #66, suggesting a shift in epitope specificity. C+ = positive control; C− = negative control. (**B**) Structural mapping of the identified epitopes onto the HER3 ECD (PDB entry: 1M6B). Left: location of peptide #54 and #55 (orange) shared by TK-A3 and TK-hu A3-H1L1. Right: location of peptide #66 (purple), recognized exclusively by TK-hu A3-H2L1. Protein domains are indicated with Roman numerals. Only HER3 domain II is rendered as surface. (**C**) Amino acid sequences of the relevant peptides with conserved regions highlighted in bold. Peptides #54 and #55 share the core motif HCFGPNPNQCC, while peptide #66 contains a distinct sequence (QPLVYNKLTFQLEPN), reflecting altered antigen recognition in TK-hu A3-H2L1.

Epitope mapping was performed using a linear peptide array to assess whether the epitopes recognized by the murine antibodies were preserved after humanization. The results showed that the humanized variant TK-hu A3-H1L1 retained specificity for the same epitope as the parental TK-A3 antibody, displaying strong binding to peptides #54 and #55 (Figure 4A, right panel), which share the conserved core sequence HCFGPNPNQCC (Figure 4B,C). This indicates that the humanization process in the H1L1 variant did not alter the original antigen-binding site.

In contrast, the TK-hu A3-H2L1 variant lost binding to peptides #55 and instead recognized peptide #66 (Figure 4A, left panel), indicating a shift in epitope specificity likely due to structural changes introduced during the humanization process. This suggests that the pairing of different heavy and light chain variants can influence antigen recognition and may result in altered binding interfaces.

Structural mapping of these epitopes onto the HER3 extracellular domain (ECD) (Figure 4B) confirmed that the epitopes recognized by TK-A3 and TK-hu A3-H1L1 (peptide #54 and #55, shown in orange) are located within domain II of HER3. The newly recognized epitope #66 (purple), targeted by TK-hu A3-H2L1 (Figure 4B), also maps to domain II, but occupies a distinct region, further supporting the observed shift in binding specificity. Finally, the epitope recognized by TK-hu A4 could not be identified using the linear peptide array, suggesting that this antibody likely binds a conformational (non-linear) epitope, consistent with previous observations for the murine TK-A4 [30].

### 3.5. Structure Determination and Epitope Characterization

The crystallographic analysis was performed using the Fab fragment derived from the TK-hu A3-H1L1 variant, selected based on its superior binding and biophysical properties. Prior to crystallization, formation of the HER3::TK-hu A3 Fab complex was verified by size-exclusion chromatography (SEC) and SDS-PAGE. SEC analysis revealed a distinct peak consistent with the expected molecular weight of the complex (~115 kDa), and SDS-PAGE of this peak confirmed the presence of both HER3 and Fab fragments. Detailed chromatographic and electrophoretic data are provided in the Supplementary Information (Supplementary Figure S1).

**Figure 5:**
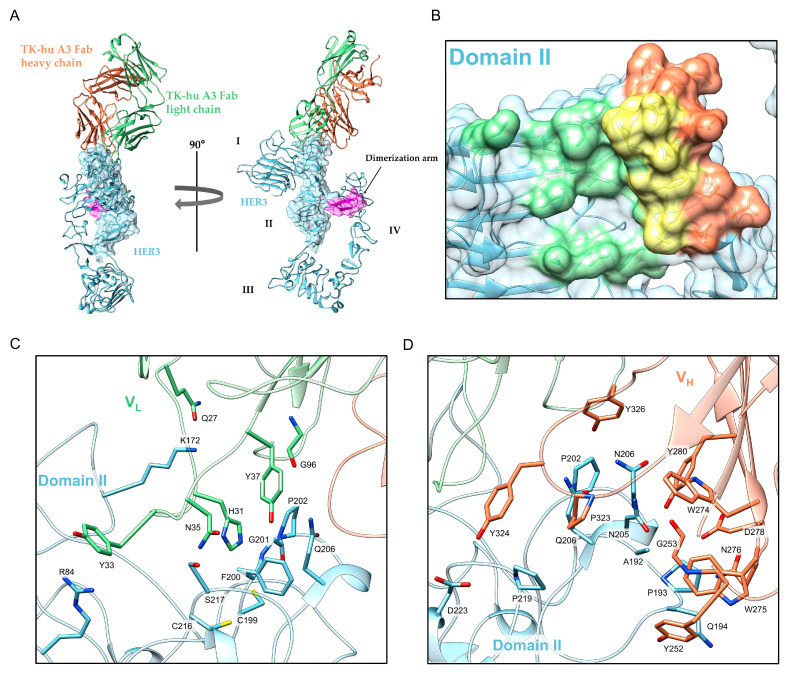
Structure of HER3::TK-hu A3 Fab complex. (**A**) Front view (left) and side view (right) of HER3 (cyan) in complex with TK-hu A3 Fab (light chain, V_L_, in light green and heavy chain, V_H_, in coral). Only HER3 domain II is rendered as surface; domains I–IV are indicated with Roman numerals. The domain II dimerization arm (residues 242–257, chain B) is shown in magenta. HER3 domain II surface colored by Fab contacts (atom–atom cutoff 4.0 Å). (**B**) Residues contacting V_L_ only are light green, residues contacting V_H_ only are coral, and residues contacting both chains are yellow. The HER3 cartoon is shown underneath (cyan); the Fab is omitted for clarity. (Contact patches computed with PDBePISA). Atomic details of HER3::TK-hu A3 Fab interface region. (**C**,**D**) TK-hu A3 Fab binds to the N-terminus region of domain II of HER3 mostly via hydrogen bonds, except for R84, which is involved in a π-cation interaction with residue Y33/F. (**C**): interactions between V_L_ (variable light chain, light green) and HER3 domain II (cyan). (**D**): interactions between V_H_ (variable heavy chain, coral) and HER3 domain II (cyan) (PDB entry: 9I1Q).

The HER3::TK-hu A3 Fab complex crystals belong to the monoclinic space group P21, with axis sizes a = 98.03 b = 98.81 c = 270.38, α = γ = 90° β = 99.21°. In the asymmetric unit, there are 4 complex molecules represented by 12 chains: chains A, B, C, and D correspond to HER3 molecules, chains E to H correspond to the light chain and chains I to L correspond to the heavy chain of TK-hu A3 Fab. Therefore, in order to achieve the main goal of this research activity, namely the identification and structural characterization of the epitope and the definition of the binding mode of TK-hu A3 Fab, we worked on one of the four complexes present in the asymmetric unit. We chose to study the complex formed by the B, F and I chains because it had lower B-factor values in the interaction region (Figure 5A). Models were least-squares superposed on HER3 Cα atoms, and pairwise Cα RMSDs (relative to chain B) over the epitope-containing segment (residues 192–219) were 1.03 Å for B–A, 1.63 Å for B–C, and 1.55 Å for B–D, indicating no meaningful structural differences at the epitope/paratope (Supplementary Figure S2). Consistently, B-factor maps of the four ASU copies, colored with the standard blue–white–red scale (range 9–262 Å^2^; white = 85 Å^2^), show the lowest interface B-factors for the B/F/I copy (Supplementary Figure S3), supporting its use as the representative model in the main text. Focusing our study on the interaction between TK-hu A3 Fab and HER3, we examined the interface using the online software PDBePISA (Proteins, Interfaces, Structures and Assemblies) [31]. Interface residues identified with PDBePISA were manually curated using an atom–atom distance baseline of ~4.0 Å. Only chemically plausible contacts were retained and well-oriented polar pairs marginally above the 4.0 Å cutoff were included when supported by geometry and density. This analysis led to important insights: both chains contribute equally to the interaction with the antigen; specifically, the light chain (F chain) interacts with a surface area of 419.3 Å^2^, while the heavy chain (I chain) interacts with a surface area of 475.9 Å^2^ (Figure 4B). The CDRs of both chains interact closely with residues located in the N-terminal region of the HER3 domain II, and examination of the interactions revealed that the HER3 residues at the interface with the TK-hu A3 Fab molecule are: R84, Q119, K172, T185, L186, T187, I190, A192, P193, Q194, C199, F200, G201, P202, N203, P204, N205, Q206, C207, C216, S217, G218, P219, Q220, D223, F225. Most of them are involved in the formation of weak bonds with Fab CDRs, described in Table 2.

In addition, residue R84 forms a π-cation interaction with residue Y33/F. Crystallographic studies confirmed the importance of certain residues for interaction (C199, F200, G201, P202, N203, N205, Q206). Furthermore, our analysis identified additional residues that contribute significantly to recognition by TK-hu A3 (Fab), including R84, K172, A192, P193, Q194, C216, S217, P219, and D223. Taken together, these results suggest that the epitope consists of residues R84, K172, A192, P193, Q194, C199, F200, G201, P202, N203, N205, Q206, C216, S217, P219, D223, and the interaction region falls at the N-terminus region of domain II of HER3 (Figure 5C,D).

Consistent with the structural interface, the epitope mapping technique described in Section 3.4, based on overlapping peptide ELISA, initially identified the sequence HCFGPNPNQCC (residues 198–208) as part of the linear epitope that falls within the crystallographic patch and overlaps the loop residues C199–Q206. Taken together, the data place the TK-hu A3 epitope on a cysteine-rich loop in HER3 domain II, adjacent to, but not overlapping, the domain-II dimerization arm (residues 242–257, Figure 5A). This geometry is consistent with steric hindrance of dimer formation and provides a structural rationale for the antibody’s inhibitory activity.

### 3.6. Native Conformation Binding and Flow Cytometry Analysis

**Figure 6:**
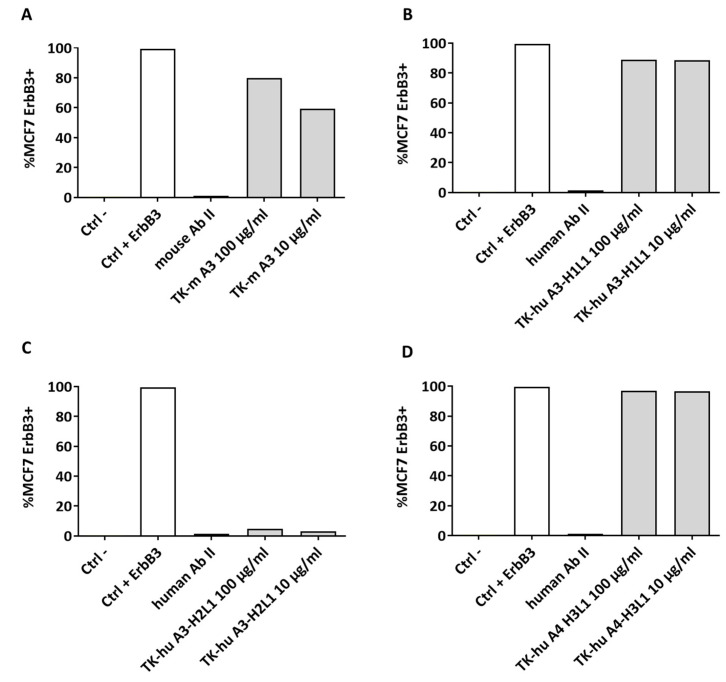
TK-hu A3 and TK-hu A4 antibodies variants bind HER3 receptor in its native conformation. MCF7 cells were incubated on ice for 1 h in the presence or absence of TK-murine A3 (**A**), TK-hu A3-H1L1 (**B**), TK-hu A3-H2L1 (**C**), and TK-hu A4-H3L1 (**D**) antibody variants at concentrations of 10 µg/mL and 100 µg/mL. After incubation, cells were washed, and antibody binding was detected using anti-mouse or anti-human IgG antibody conjugated to Alexa Fluor 488 (gray bars). Cells were analyzed using a CytoFLEX flow cytometer platform. Histograms display the percentage of HER3-positive cells. CTRL antibody: The APC anti-human HER3/HER-3 Antibody was used as a positive control (white bars).

The ability of the humanized antibodies to bind to HER3 in its native conformation was assessed using flow cytometry. MCF7 cells were incubated with the antibody variants, and their binding was analyzed. Both the murine and humanized TK-hu A3-H1L1 and TK-hu A4-H3L1 antibodies displayed efficient binding to HER3, as evidenced by the high percentage of positive cells (Figure 6A,B,D). The TK-hu A3-H2L1 variant failed to bind to the HER3 receptor on MCF7 cells (Figure 6C), leading to its exclusion from further studies. Based on these findings, both TK-hu A3-H1L1 and TK-hu A4-H3L1 were carried forward for further evaluation in vitro and in vivo.

### 3.7. Binding Affinity

The binding affinity of the humanized and murine antibodies to HER3 was further analyzed using surface plasmon resonance (Biacore technology). The K_D_ values indicated that TK-murine A3, TK-hu A3-H1L1, and TK-hu A4-H3L1 antibodies all had significantly high affinity for HER3 (Table 3). However, the sensorgrams for murine TK-A4 displayed a non-classical shape, suggesting a non-canonical binding profile. One possible explanation is antibody self-aggregation, which may influence interaction stoichiometry in Biacore assays. Alternatively, TK-A4 may recognize a conformational epitope that requires specific structural conditions for stable binding, as suggested by our inability to map its epitope using a linear peptide library. While these issues did not prevent HER3 binding in ELISA or flow cytometry, the associated variability contributed to the decision to deprioritize TK-A4 and TK-hu A4 for further development.

### 3.8. Mechanism of Action of TK-hu A3 and TK-hu A4 Antibodies

**Figure 7:**
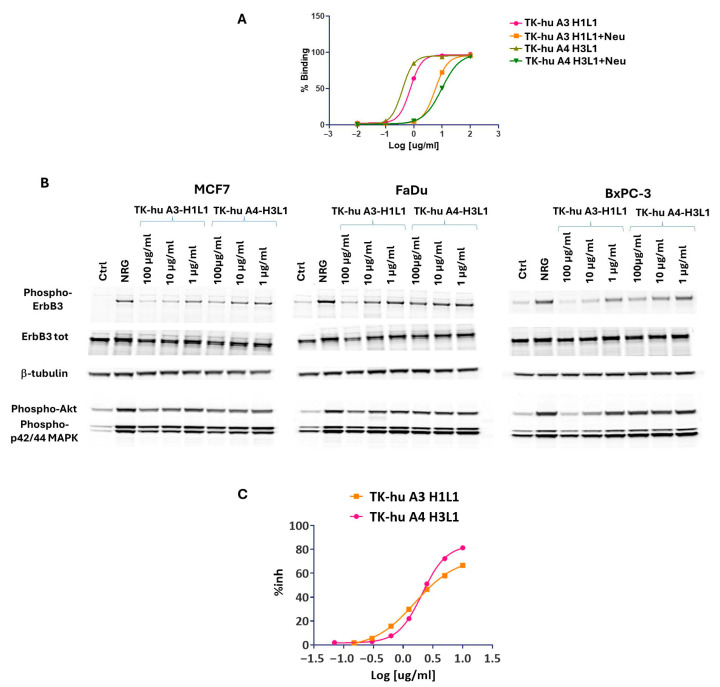
TK-hu A3 and TK-hu A4 antibodies inhibit NRG-driven HER3 signaling and reduce cancer cell viability. (**A**) Neuregulin competition assay. SK-BR-3 cells were incubated for 1 h at 4 °C with increasing concentrations of TK-hu A3 or TK-hu A4, either in the presence or absence of recombinant neuregulin (NRG). Binding to cell-surface HER3 was assessed by flow cytometry. The plot shows representative binding curves: TK-hu A3 ± NRG (purple and yellow), and TK-hu A4 ± NRG (light green and dark green). The percentage of HER3-positive cells is plotted on the y-axis versus antibody concentration (log10 [µg/mL], x-axis). (**B**) Western blot analysis of HER3 signaling pathway activation in MCF7, FaDu, and BxPC-3 cells. Cells were pretreated for 6 h with increasing concentrations of TK-hu A3 or TK-hu A4 antibodies, then stimulated with NRG. The Control (Ctrl) lane represents untreated, unstimulated cells, while the NRG lane represents NRG-stimulated cells in the absence of antibody. Blots were probed for phosphorylated HER3 (pHER3, Tyr1289), total HER3, phosphorylated Akt (pAkt, Ser473), and phosphorylated p42/44 MAPK (Thr202/Tyr204). β-tubulin served as the loading control. (**C**) Colony-formation assay. BxPC-3 cells were seeded in 6-well plates, treated or not with increasing concentrations of TK-hu A3-H1L1 or TK-hu A4-H3L1 and cultured to allow colony formation; colonies were then fixed, stained, and counted. Only colonies comprising >50 cells were scored as survival colonies. The percentage of inhibition of colony formation (% inh) relative to untreated controls is plotted against the logarithmic antibody concentration (log_10_ [µg/mL]), and data are fitted with a four-parametric non-linear regression curve (GraphPad v8.0).

To thoroughly investigate the mechanisms by which TK-hu A3 and TK-hu A4 exert their effects, we conducted a series of experiments including a NRG competition assay, pathway inhibition analysis, and colony-formation assays. These comprehensive analyses aimed to elucidate how these antibodies interfere with cancer cell survival and proliferation. Antibody binding to overexpressed HER family receptors (HER1, HER2, HER3) on tumor cells interferes with ligand binding or inhibits their homo- and hetero-dimerization with other HER family members. This interference inhibits the activation of downstream MAPK/ERK and PI3K/AKT signaling pathways that promote tumor cell growth, migration, and proliferation. Inhibition of the downstream pathway RAF/MAPK/ERK has been largely associated with reduced tumorigenesis, as these pathways govern fundamental physiological processes such as cell proliferation, differentiation, metabolism, and cytoskeleton reorganization [19]. The PI3K/Akt/mTOR pathway is known to lead to tumorigenesis via increased transcription and cellular proliferation. First, since NRG is one of the main ligands for HER3, we tested whether TK-hu A3 and TK-hu A4 can compete with NRG for binding to the HER3 receptor. SK-BR-3 cells expressing HER3 were treated with increasing concentrations of TK-hu A3 or TK-hu A4 for 1 h at 4 °C in the presence of NRG. The experiment showed that high-affinity NRG binding is completely displaced by both antibodies (Figure 7A).

To further understand the impact of TK-hu A3 and TK-hu A4 on ligand-driven HER3 signaling, we performed Western blot analyses using HER3-expressing cancer cell lines (MCF7, FaDu, BxPC-3) stimulated with NRG following antibody treatment. Cells were pretreated with increasing concentrations of either antibody variant for 6 h, followed by NRG stimulation. Protein lysates were analyzed for the activation status of HER3 and downstream targets, including Akt and p42/44 MAPK, using phospho-specific antibodies (Figure 7B).

Pretreatment with both TK-hu A3 and TK-hu A4 antibodies resulted in inhibition of pHER3 (Tyr1289) in a dose-dependent manner. Similarly, decreased phosphorylation of Akt (Ser473) and MAPK (Thr202/Tyr204) was also observed in the three cell lines, particularly in BxPC-3 and FaDu cells. Notably, the inhibitory effect of TK-hu A3 appeared more pronounced than TK-hu A4 across all three cell lines, with the strongest suppression of pHER3, pAkt, and pMAPK levels in BxPC-3 cells.

To quantify the extent of HER3 pathway suppression, densitometric analysis of the Western blot bands from Figure 7B was performed and presented in Supplementary Figure S4. Raw integrated density (RawIntDen) values were obtained for each phosphorylated marker across treatments and cell lines. The results confirmed a dose-dependent inhibition of HER3 signaling, especially in BxPC-3 cells, which demonstrated the highest sensitivity to TK-hu A3. These findings support the functional capability of both antibody variants to disrupt HER3-mediated signaling cascades, with TK-hu A3 showing superior potency.

To test the hypothesis further, a colony-formation assay was performed. As expected, a significant inhibition of cell survival was observed in BxPC-3 cells treated with TK-hu A3 and TK-hu A4, confirming the impact of these antibodies on cancer cell viability (Figure 7C).

### 3.9. In Vivo Antitumor Potential of TK-hu A3

**Figure 8:**
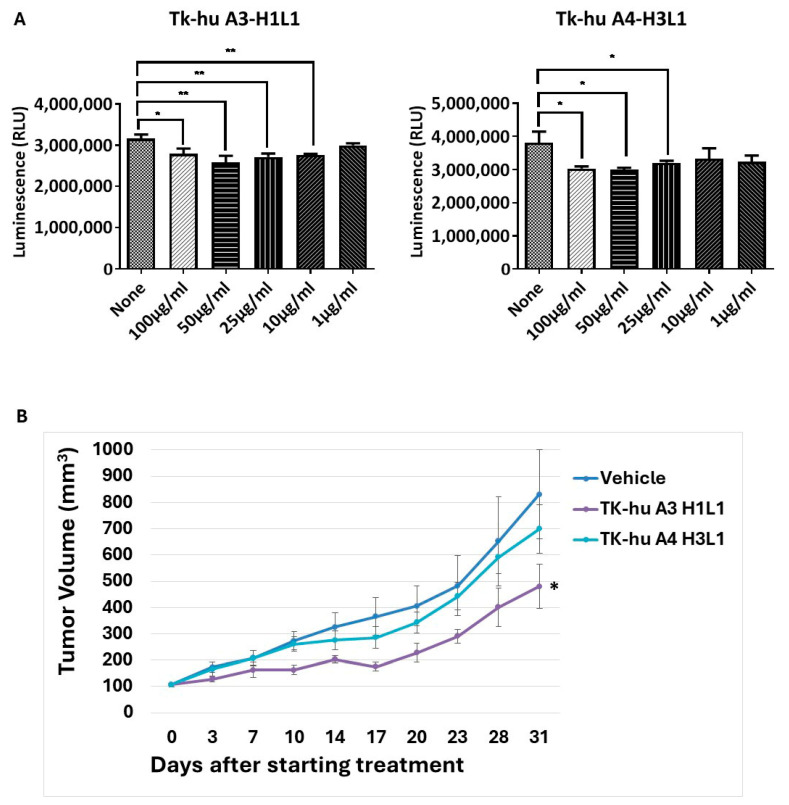
Ability of TK-hu A3-H1L1 to control cancer cell growth in vitro and in vivo. (**A**) In vitro cell proliferation assay. BxPC-3 pancreatic cancer cells were treated with different concentrations of TK-hu A3-H1L1 and TK-hu A4-H3L1 antibodies indicated on the x-axis. Following 72 h, the luminescence (Relative Light Units, RLU) was recorded. The number of viable cells was directly proportional to ATP concentration. (**B**) In vivo efficacy in a pancreatic cancer xenograft model. BxPC-3 cells were subcutaneously implanted into immunodeficient nude mice. Once tumors reached an average volume of 100 mm^3^, animals were randomized into treatment groups (n = 8 mice per group) and administered with intraperitoneal injections of TK-hu A3 or TK-hu A4 (20 mg/kg, twice weekly). Control mice received vehicle only. The results are expressed as mean tumor volume (mm^3^) ± SEM. * *p* < 0.05, ** *p* < 0.005.

To translate the results obtained in vitro into potential pre-clinical relevance, we tested the therapeutic efficacy of TK-hu A3 and TK-hu A4 in vivo, in a mouse xenograft model of pancreatic cancer. Initially, the ability of TK-hu A3 and TK-hu A4 to impair cancer cell growth was tested in vitro using proliferation assays. In these assays, BxPC-3 cells were treated with different concentrations of TK-hu A3 and TK-hu A4, and after 72 h, the number of metabolically active cells was measured. The results demonstrated a significant reduction in cell proliferation in TK-hu A3 and TK-hu A4 treated cells compared to untreated controls (Figure 8A).

Based on these promising in vitro findings, BxPC-3 cells were inoculated into nude mice, and treatment with TK-hu A3 and TK-hu A4 commenced once tumors reached an average size of 100 mm^3^. Mice were intraperitoneally injected with 20 mg/kg of TK-hu A3 or TK-hu A4, twice a week, until control tumors reached an average size of 1500 mm^3^. As shown in Figure 8B, tumor growth was significantly delayed in the group of mice receiving TK-hu A3, highlighting its potential as an effective anti-tumor agent. Based on these results, TK-hu A3 was selected for further development and proceeded to toxicology evaluation, whereas TK-hu A4 was not included in subsequent preclinical safety studies.

### 3.10. Toxicology Study

To evaluate the safety profile of TK-hu A3-H1L1, a toxicity study was conducted in rats. Six male and six female rats were treated with a single dose of TK-hu A3-H1L1 at 20, 40, or 80 mg/kg, administered intraperitoneally (i.p.). A control group, consisting of three animals per gender, received a single i.p. injection of the vehicle (sterile PBS, pH 7.4). Clinical observations, including mortality, body weight, and any signs of toxicity, were monitored throughout the study. On day 5, all animals were subjected to hematochemical analysis, followed by post-mortem pathological examinations.

No mortality occurred during the study, and no clinical signs or behavioral changes were observed in either sex for the entire study duration. Regarding body weight, no variation was observed compared to the control group in either sex. Hematology analysis revealed a slight decrease in WBC count in the treated females, particularly at the lower dose. Moreover, a limited and non-dose-related increase in some elements of the leukocyte differential count (namely neutrophils and monocytes) was registered starting from the low dose in both sexes, suggesting a minimal inflammatory response. Additionally, biochemical analysis, consistent with hematological data, showed a slight increase in globulin content at intermediate and high dosages in male and female subjects. The remaining parameters analyzed did not show any appreciable variation. Upon post-mortem examination, on day 5 after dosage, overall pathology and organ weights showed no significant variations, consistent with hematological data. Alterations recorded were: (1) signs of reactive lymphadenopathy in cervical lymph nodes in the high-dose group; (2) minimal increase in thymus; (3) minimal increase in spleen weight in the high-dose male group and in the intermediate and high-dose female group; (4) decrease in thymus and spleen weights in the low-dose female group, consistent with the respective decrease in WBC values.

Overall, the toxicology study demonstrated that TK-hu A3-H1L1 was well-tolerated at the doses tested, with no significant adverse effects on clinical signs, body weight, or major organ pathology. The observed minor alterations, such as changes in leukocyte counts and slight increases in spleen and thymus weights at higher doses, were minimal and non-dose-related, suggesting a favorable safety profile for TK-hu A3-H1L1. These findings support the continued development of TK-hu A3-H1L1 as a therapeutic candidate, with further studies warranted to explore its long-term safety and efficacy.

## 4. Discussion

In this study, we successfully humanized two murine monoclonal antibodies, TK-A3 and TK-A4, targeting the HER3 receptor, which plays a pivotal role in the progression and therapeutic resistance of various cancers. The antibodies TK-hu A3 and TK-hu A4 were developed from murine counterparts that were obtained through a specific immunization protocol. This involved immunizing mice using the electro gene transfer (EGT) method to deliver a vector encoding the HER3 antigen. This innovative approach effectively induces a robust immunological response against the HER3 protein, essential for the generation of monoclonal antibodies with high specificity. This method of immunization has been validated in previous studies, demonstrating the efficacy of electro gene transfer in eliciting potent immune responses, which are crucial for developing effective therapeutic antibodies [30,32]. These foundational studies underscore the robustness of the immunization approach and its significance in producing antibodies that maintain a high binding affinity and specificity for the HER3 receptor.

Our findings demonstrate that the humanized version of TK-A3; i. retains its specificity for HER3, ii. effectively competes with the ligand NRG, iii. inhibits downstream signaling pathways, and iv. exhibits significant antitumor activity in vivo. The specificity of TK-hu A3 and TK-hu A4 for HER3 was confirmed through ELISA, showing no cross-reactivity with other members of the ErbB family. This high specificity is critical for minimizing off-target effects, which is a significant challenge in the development of targeted therapies [33,34]. By integrating peptide mapping with X-ray crystallography, we located the TK-hu A3 epitope on a cysteine-rich loop in the N-terminal region of domain II of the HER3 ectodomain. In the crystal structure, loop residues C199, F200, G201, P202, N203, N205, Q206 make direct contacts with the Fab, with additional neighboring contacts (R84, K172, A192, P193, Q194, C216, S217, P219, D223; see Table 2). This epitope is adjacent to, but does not overlap, the domain-II dimerization arm (residues 242–257 in our numbering). Independently, peptide-array mapping identified a linear core (198–208; HCFGPNPNQCC) that falls within this interface and overlaps the loop contacts seen crystallographically, providing orthogonal support for the epitope assignment. The proximity of the epitope to the dimerization arm offers a structural rationale for steric hindrance of domain-II–mediated dimerization [35], consistent with the observed inhibition of NRG-driven signaling and in vivo activity. Over the past decade, several anti-HER3 antibodies have been developed, many of which target the ligand-binding domains of the receptor [36]. Among these, patritumab (U3-1287/AMG 888) progressed furthest in clinical development, including as an ADC. Although patritumab lacks a resolved crystal structure, preclinical and mechanistic studies strongly suggest that it binds the extracellular domain of HER3 within the ligand-binding interface. Specifically, functional studies have shown that patritumab competes with NRG for HER3 binding, thereby inhibiting ligand-induced HER3 phosphorylation and downstream signaling [37]. In contrast, TK-hu A3 recognizes a conformational epitope distinct from the ligand-binding region. By targeting domain II, TK-hu A3 can simultaneously interfere with ligand binding and receptor heterodimerization, offering a potential mechanistic advantage over patritumab’s mode of action.

TK-hu A3 and TK-hu A4 antibodies were also able to compete with NRG for HER3 binding, effectively blocking the activation of key signaling pathways, such as PI3K/Akt/mTOR and MAPK/ERK. These pathways are well-documented for their roles in promoting cell proliferation, survival, and metastasis in various cancers [38,39,40]. The ability of TK-hu A3 to inhibit these pathways suggests that it can effectively disrupt the signaling cascades essential for tumor growth and progression. The in vitro assays, including colony-formation and Western blot analyses, showed that TK-hu A3 and TK-hu A4 could significantly inhibit cancer cell survival and reduce the phosphorylation of key signaling molecules. However, it was in the in vivo setting that TK-hu A3 distinguished itself as the more potent therapeutic candidate. The delayed tumor growth observed in the TK-hu A3-treated group of mice indicates that this antibody could exert a substantial therapeutic effect in HER3-driven cancers. The failure of TK-hu A4 to achieve similar in vivo efficacy, despite its performance in vitro, underscores the complexity of translating in vitro findings to in vivo contexts. This discrepancy might be attributed to differences in pharmacokinetics, biodistribution, or the stability of the antibody in the physiological environment [41]. Further investigation into the pharmacological properties of these antibodies could provide insights into optimizing their therapeutic potential.

HER3 has emerged as a critical mediator of resistance to therapies targeting other members of the ErbB family, including EGFR and HER2 inhibitors [42,43,44]. The ability of TK-hu A3 to specifically target HER3 and inhibit its signaling pathways positions it as a promising candidate for combination therapies aimed at overcoming resistance to existing treatments. Moreover, by targeting domain II, which plays a central role in mediating dimerization with other ErbB receptors, TK-hu A3 may effectively block coreceptor engagement and disrupt signal transduction [45]. For example, TK-hu A3 could potentially be combined with EGFR or HER2 inhibitors to enhance therapeutic efficacy and prevent the emergence of resistant cancer cell populations. In addition to its direct antitumor effects, the humanized antibody described here could also be developed as a versatile platform for other therapeutic modalities. TK-hu A3 could be engineered as a T-cell engager, antibody-drug conjugate (ADC), or chimeric antigen receptor T-cell (CAR-T) therapy. Notably, HER3-targeted ADC has shown promising clinical results, demonstrating potent antitumor activity in patients with HER3-expressing cancers [46,47,48,49]. In support of its potential use as an antibody-drug conjugate (ADC), we have also observed that TK-hu A3 undergoes efficient internalization in HER3-expressing cancer cells, a critical requirement for intracellular drug delivery. Furthermore, the potential application of TK-hu A3 as bispecific antibody, targeting both HER3 and EGFR, could offer a novel approach to simultaneously inhibit multiple pathways involved in tumor growth and resistance [50]. By harnessing these approaches, TK-hu A3 could be further optimized to enhance its efficacy and broaden its therapeutic applications.

The safety profile of TK-hu A3, as observed in the toxicology study, further supports its potential for clinical development. The minimal inflammatory responses and lack of significant organ toxicity at therapeutic doses are encouraging, suggesting that TK-hu A3 could be well-tolerated in clinical settings. However, further studies, including dose-escalation trials and long-term safety assessments, are necessary to fully establish its safety profile.

## 5. Conclusions

In summary, TK-hu A3 has demonstrated strong potential as a therapeutic agent for HER3-driven cancers, particularly in the context of resistance to other ErbB-targeted therapies. Beyond its role as a standalone therapeutic, TK-hu A3 could also serve as the basis for novel treatment strategies, including T-cell engagers, ADCs, CAR-T therapies, and bispecific antibodies. The development of TK-hu A3 could represent a significant advancement in the treatment of cancers with high HER3 expression and resistance to conventional therapies, warranting further preclinical and clinical investigation.

## Figures and Tables

**Table 1:** Data collection and refinement statistics for the HER3::TK-hu A3 (Fab) Complex (PDB entry: 9I1Q). Values in parentheses correspond to the high-resolution shell.

HER3::Tk-hu A3 (Fab)	
Data Collection	
Space Group	P2_1_
Unit-cell dimension (Å)	a = 98.03; b = 98.81; c = 270.38 α = γ = 90.00°; β = 99.21°
Resolution range (Å)	96.76–2.55 (2.94–2.55)
Number of observations	540,804 (26,627)
Unique reflections	82,868 (4143)
Completeness (%) (spherical)	49.9 (7.2)
Completeness (%) (ellipsoidal)	87.6 (63.7)
Redundancy	6.5 (6.4)
Ι/σ (I)	12.3 (1.9)
R_merge_ (%) **^a^**	12.0 (102.9)
CC_1/2_	0.999 (0.649)
Wilson B-value (Å^2^)	58.0
Refinement	
R_work_ **^b^**/R_free_ **^c^** (%)	21.1/26.0
Average B, all atoms (Å^2^)	71.0
r.m.s.d. bond (Å)	0.007
r.m.s.d. angles (°)	1.130
Ramachandran (%) Favored/allowed/outliers	94/6/0
Total number of atoms	31,872
Water molecules	105

**a.** Rmerge = Σh Σl |Ihl − <Ih>|/ Σh Σl <Ih>, where Ihl is the lth observation of reflection h and <Ih> is the weighted average intensity for all observations l of reflection h. **b.** Rwork factor = Σh||Fobs(h)| − |Fcal(h)||/Σh|Fobs(h)|, where Fobs(h) and Fcal(h) are the observed and calculated structure factors for reflection h, respectively. **c.** Rfree factor was calculated the same as Rwork factor using the 5% the reflections which were selected randomly and omitted from refinement.

**Table 2:** HER3::TK-hu A3 Fab interface residues. HER3 (chain B) residues contacting the Fab are listed by interaction type. Contacts were identified manually using a ~4.0 Å distance cutoff and curated for chemical plausibility. “/F” and “/I” indicate V_L_ (chain F) and V_H_ (chain I), respectively. The listed HER3 residues defined the structural epitope.

Hydrogen Bonds		Hydrophobic Interactions	
HER3-ECD	TK-hu A3 Fab	HER3-ECD	TK-hu A3 Fab
K172	Q27/F	R84	Y33/F
A192	Y280/I		
P193	D278/I		
Q194	N276/I, W275/I, Y252/I		
C199	H31/F		
F200	Y37/F, N35/F		
G201	H31/F		
P202	G96/F, Y280/I		
N203	W274/I, Y326/I		
N205	G253/I, W274/I		
Q206	Y37/F, P323/I		
C216, S217	N35/F		
P219	Y234/I		
D223	Y324/I		

**Table 3:** Measurement of binding affinity for HER3. The affinity of TK-A3, TK-A4, TK-hu A3 (H1L1), and TK-hu A4 (H3L1) for the HER3 Fc chimera protein was calculated by surface plasmon resonance detection using a Biacore X100 instrument (*k_off_* antibody dissociation rate; *k_on_* antibody association rate; K_D_ = *k_off_/k_on_*).

Antibody	*k_on_* (1/Ms)	*k_off_* (1/s)	K_D_ (M)
TK-A3	3.83 × 10^4^	8.17 × 10^−5^	2.13 × 10^−9^
TK-A4	* NA	* NA	* NA
TK-hu A3	6.24 × 10^4^	1.13 × 10^−4^	1.81 × 10^−9^
TK-hu A4	3.39 × 10^5^	1.64 × 10^−3^	4.84 × 10^−9^

* NA: Not applicable.

## Data Availability

The data and materials are available from the corresponding author upon reasonable request.

## Supplementary Materials

The following supporting information can be downloaded at: https://www.mdpi.com/article/10.3390/antib14040084/s1, Figure S1: HER3::TK-hu A3 (Fab) complex purification. (A) Size-exclusion chromatography; Figure S2: Copy-to-copy superposition of the four HER3::TK-hu A3 Fab complexes in the asymmetric unit [28]; Figure S3: B-factor maps at the HER3–Fab interface for the four ASU copies (same field of view used in Figure S2); Figure S4: Raw band intensity measurements of phosphorylated signaling proteins in response to antibody treatment.

## Abbreviations

The following abbreviations are used in this manuscript:

RTKs	Receptor tyrosine kinases
TKis	Small-molecule tyrosine kinase inhibitors
FBS	Fetal Bovine Serum
FCS	Fetal calf serum
FGFR2	Fibroblast growth factor receptor 2
PBS	Phosphate-Buffered Saline
ATCC	American Type Culture Collection
siRNA	Small-interfering RNA
AP	Alkaline Phosphatase
APC	Allophycocyanin
NRG	Neuregulin
ASU	Asymmetric unit
