# Supplementary material for: Novel Humanized Anti-HER3 Antibodies: Structural Characterization and Therapeutic Activity
Source: Antibodies (Basel). 2025 Oct 6;14(4):84. doi: 10.3390/antib14040084 (PMC12550933; doi:10.3390/antib14040084)
Supplement: Supplementary file 1 [file antibodies-14-00084-s001.zip › antibodies-3767488-supplementary.pdf]

To assess crystallization trials, we proceeded to form the HER3::TK-hu A3 (Fab) complex. The deglycosylated HER3 protein and purified Fab of the TK-hu A3 antibody were mixed in a 1:2 stoichiometric ratio, with a stoichiometric excess of Fab. The complex was then injected into a Superdex 200 increase 10/300 GL column for size-exclusion chromatography. The chromatographic profile showed three peaks, which were collected in seven fractions: the first peak eluted at 7.5 ml (fraction 1, indicated by the black arrow in figure S1A), the second one eluted at 9-10 ml (fraction 2-4, indicated by the red arrow in figure S1A), and the last one eluted at approximately 15 ml (fraction 5-7, indicated by the green arrow in figure S1A). We analyzed the collected fractions by SDS-PAGE, obtaining the following results: fractions 2-4 contain both HER3 (~65 kDa) and TK-hu A3(Fab) (~50 kDa) (Figure S1B, lanes 2-4), confirming the formation of the complex (~115 kDa), while the fraction 1, isolated from the first peak, contains aggregated HER3 and does not present Fab (Figure S1B, lane 1). Fractions 6 and 7, on the other hand, correspond to the Fab excess (Figure S1B, lanes 6 and 7). Fractions 2-4 were pooled together and concentrated to a final concentration of 10 mg/ml for crystallization tests.

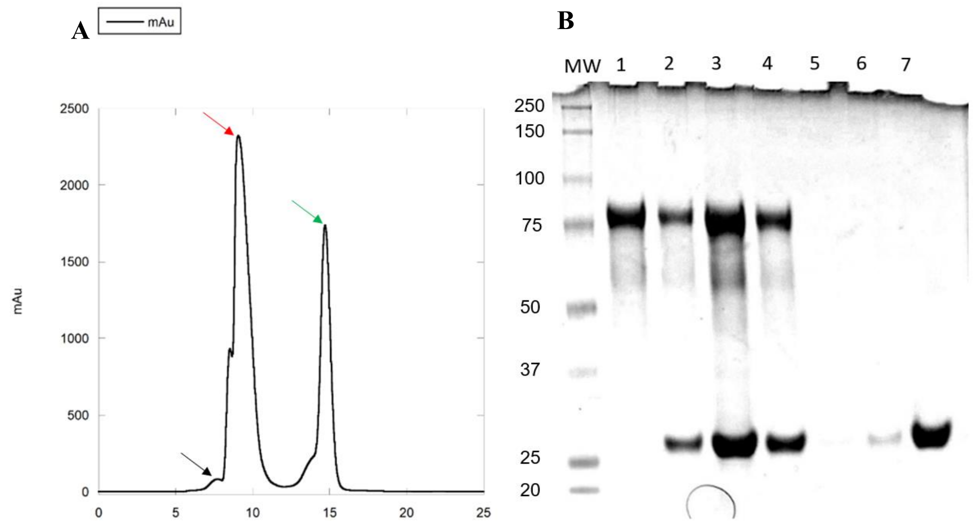

Figure S1. HER3::TK-hu A3 (Fab) complex purification. (A) Size-exclusion chromatography. The first peak indicated by a black arrow is due to HER3 aggregation at high concentration, the second peak indicated by a red arrow corresponds to the complex, while the third peak at the TK-hu A3 Fab excess (green arrow). X-axis: elution volume (ml); Y-axis: absorbance (mAU). **(B) SDS-PAGE.** In order from left to right: molecular weight (MW), fraction corresponding to the HER3 aggregation peak (1), fractions corresponding to the complex peak (2-4), fractions corresponding to the TK-hu A3 Fab excess peak (5-7).

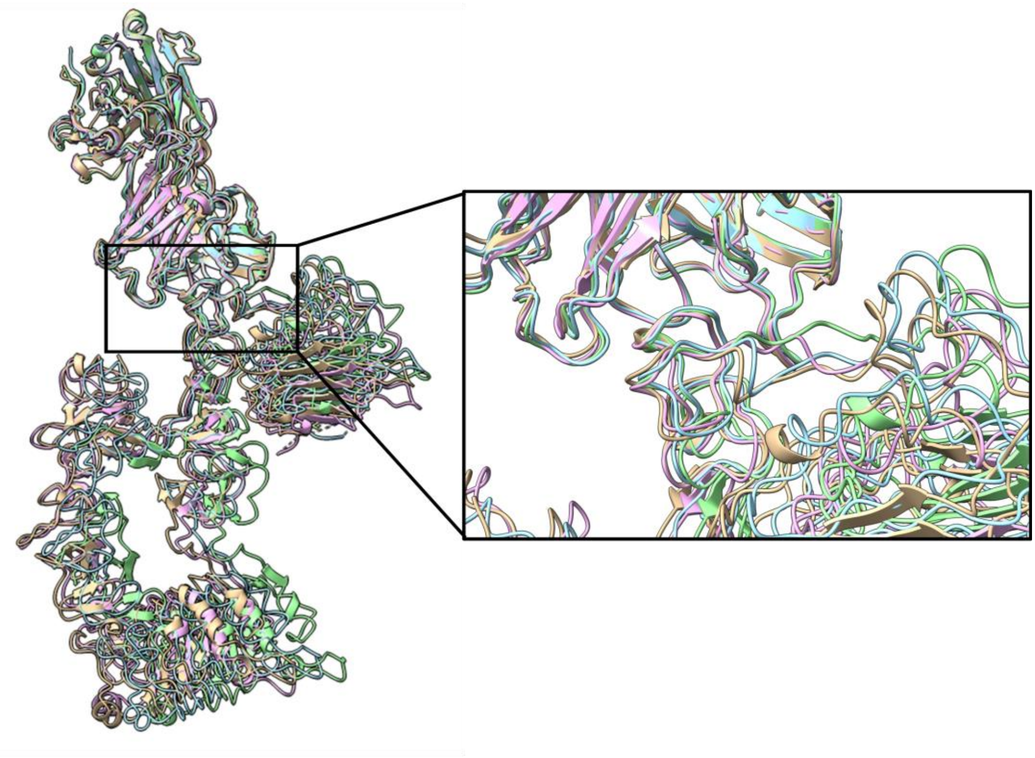

Figure S2. Copy-to-copy superposition of the four HER3::TK-hu A3 Fab complexes in the asymmetric unit. Left, global overlay; right, zoom on the HER3 domain-II epitope interface. For readability, each complex (HER3 + TK-hu A3 Fab) is colored uniformly: sand = B/F/I, lavender = A/L/H, light green = C/E/J, light blue = D/G/K. A-D HER3 chains; E-H: TK-huA3 Fab light chains; H-K: TK-huA3 Fab heavy chains. Models were least-squares aligned on HER3 Cα atoms; pairwise Cα RMSDs over the epitope-containing segment (residues 192–219) relative to chain B are 1.03 Å (B–A), 1.63 Å (B–C), and 1.55 Å (B–D), indicating no meaningful structural differences among asymmetric unit (ASU) copies. RMSD values were calculated with UCSF ChimeraX v1.9 [28].

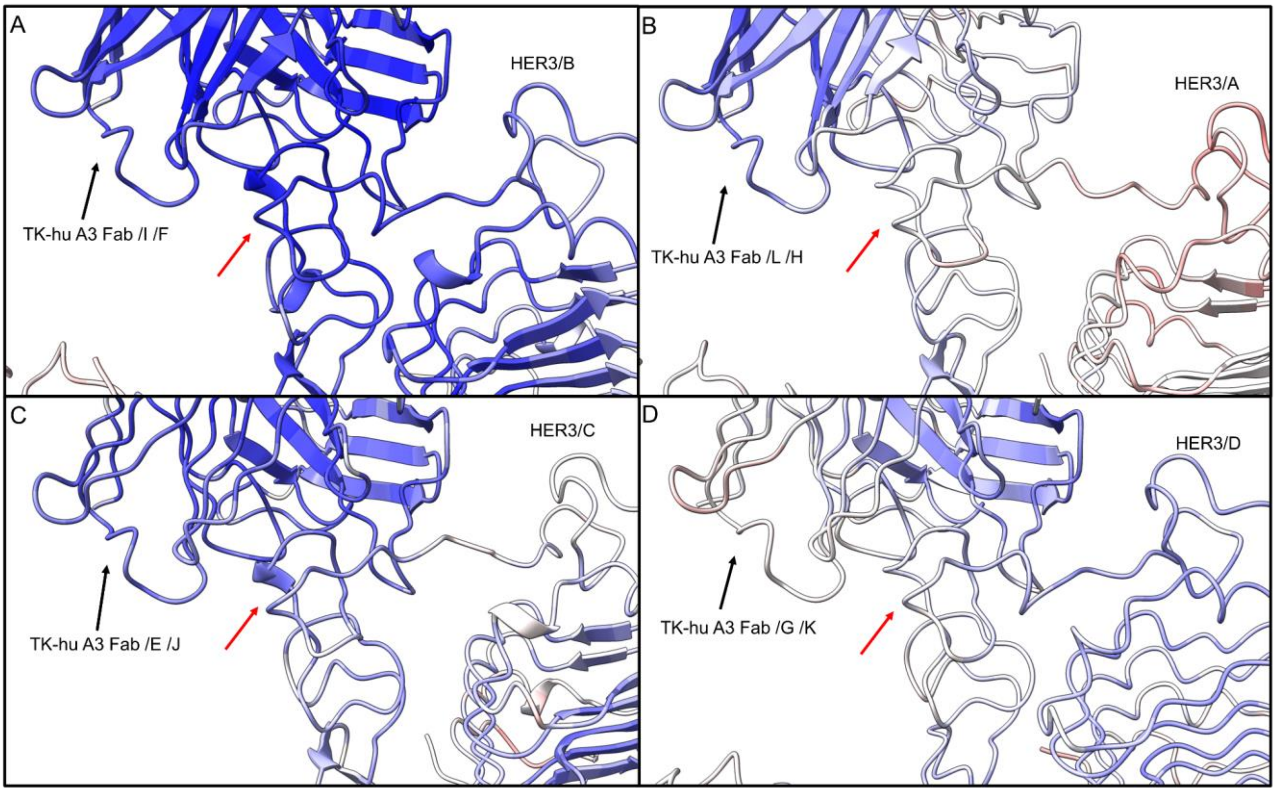

Figure S3. B-factor maps at the HER3–Fab interface for the four ASU copies (same field of view used in Figure S2). Cartoon/atoms are colored by B-factor using the standard blue–white–red palette (range 9 - 262 Å^2, white = 85 Å^2): blue = lower B (better defined), red = higher B (more disordered). The B/F/I copy shows the lowest interface B-factors and was used as the representative model in the main text. A red arrow indicates the epitope region. B-factor values were calculated with UCSF ChimeraX v1.9.

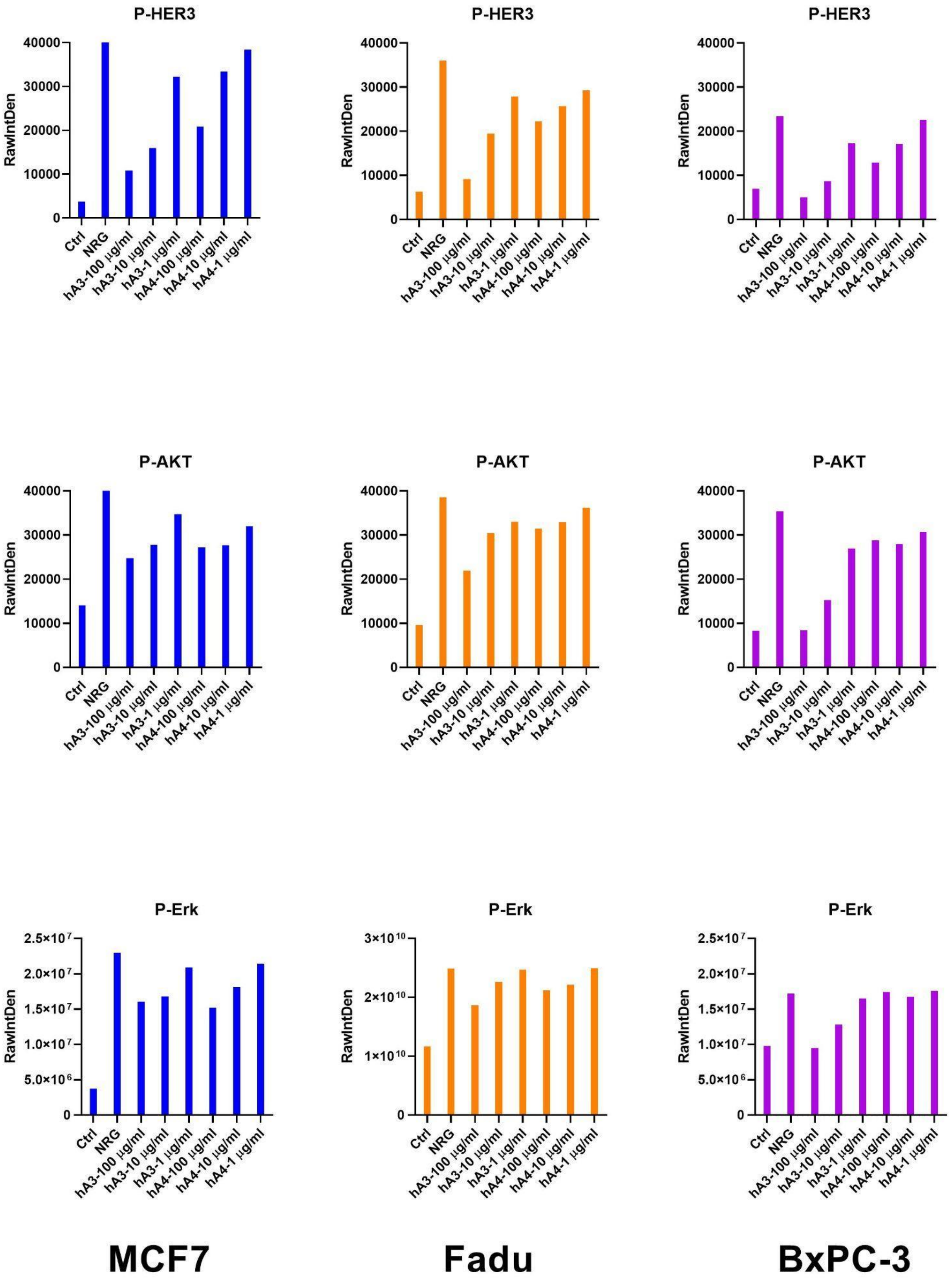

Figure S4. Raw band intensity measurements of phosphorylated signaling proteins in response to antibody treatment**.** Densitometric analysis of Western blot bands from Figure 7B was performed using ImageJ, and values are reported as Raw Integrated Density (RawIntDen) for p-HER3, p-AKT, and p-ERK. Band intensities were measured across three HER3-expressing tumor cell lines: MCF7 (blue), FaDu (orange), and BxPC3 (violet), following Neuregulin-1 (NRG) stimulation

and treatment with TK-hu A3 or TK-hu A4 antibody variants. The plots reflect direct intensity values of phosphorylated protein bands and illustrate inhibition of HER3 pathway activation in response to antibody treatment.

References

28. Meng EC, Goddard TD, Pettersen EF, Couch GS, Pearson ZJ, Morris JH, et al. UCSF ChimeraX: Tools for structure building and analysis. *Protein Sci*. **2023** Nov;32(11):e4792.
